# Modern reproductive patterns associated with estrogen receptor positive but not negative breast cancer susceptibility

**DOI:** 10.1093/emph/eou028

**Published:** 2014-11-11

**Authors:** C. Athena Aktipis, Bruce J. Ellis, Katherine K. Nishimura, Robert A. Hiatt

**Affiliations:** ^1^Center for Evolution and Cancer, University of California San Francisco, 2340 Sutter Street S-341, Box 0128, San Francisco, CA 94143-0128, USA; ^2^Department of Psychology, Arizona State University, PO Box 871104, Tempe, AZ 85287-1104, USA; ^3^Norton School of Family and Consumer Sciences, University of Arizona, 650 N Park Ave, Tucson, AZ 85721, USA; ^4^Department of Epidemiology and Biostatistics, University of California San Francisco, Box 0560, UCSF, San Francisco, CA 94143, USA

**Keywords:** evolutionary mismatch, breast cancer heterogeneity, cancer evolution, hormone-associated breast cancer, parity, age of first birth

## Abstract

It has long been accepted that modern reproductive patterns are likely contributors to breast cancer susceptibility because of their influence on hormones such as estrogen and the importance of these hormones in breast cancer. We conducted a meta-analysis to assess whether this ‘evolutionary mismatch hypothesis’ can explain susceptibility to both estrogen receptor positive (ER-positive) and estrogen receptor negative (ER-negative) cancer. Our meta-analysis includes a total of 33 studies and examines parity, age of first birth and age of menarche broken down by estrogen receptor status. We found that modern reproductive patterns are more closely linked to ER-positive than ER-negative breast cancer. Thus, the evolutionary mismatch hypothesis for breast cancer can account for ER-positive breast cancer susceptibility but not ER-negative breast cancer.

## INTRODUCTION

It has long been known that breast cancer is associated with reproductive factors such as age of menarche, parity and reproductive timing [1]. However, breast cancer is a heterogeneous disease, and different subtypes of breast cancer have different risk factors [2–11]. In this review, we take an evolutionary approach to examine how risk factors associated with modern reproductive patterns as opposed to those characteristic of ancestral peoples differ with regard to estrogen receptor (ER) status.

Evolutionary approaches to health and medicine have become increasingly prevalent over the last two decades, [12–14], with more attention being paid to evolutionary theory and methods in varied areas of medicine. Evolutionary theory applies to many aspects of cancer [15–18], including the role of modern environments in shaping susceptibility to cancer. ‘Evolutionary mismatch’ between ancestral and modern conditions plays a role in a variety of diseases including cancer [19].

### Evolutionary mismatch theory and breast cancer susceptibility

When environments change rapidly, natural selection may be too slow to adapt our phenotypes to the new condition. The resulting mismatch can have various consequences, including dysregulation of cancer suppression mechanisms that increase vulnerability to cancer. A variety of modern ecological, demographic and cultural changes appear to contribute to cancer risk, including increased nutrition [20], changes in reproductive patterns [21–23], population migrations [24] and changes in cultural practices such as smoking [25]. In this article, we focus on the association of breast cancer and modern reproductive patterns, which are characterized by earlier age at menarche, delayed reproduction and lower fertility than would have been the case for ancestral humans [21–23]. Here we define modern societies as any societies’ post-demographic transition, where there are both low birth rates and death rates.

Previous researchers have proposed that high rates of breast cancer in the modern world result, in part, from a modern reproductive pattern, with women experiencing on the order of 300–400 menstrual cycles while our ancestors were likely to have experienced 100 or less [21–23]. According to this view, the large increase in number of cycles in modern humans causes higher levels of cyclic hormone exposure over the lifetime that made modern women more susceptible to breast cancer than our pre-agricultural ancestors. This aspect of breast cancer etiology is generally accepted among epidemiologists [26, 27], and it is consistent with established and commonly accepted risk factors for breast cancer such as early menarche, low fertility, later age of first birth and later menopause [28]. In this article, we test whether mismatch between ancestral and modern reproductive patterns is consistent with breast cancer susceptibility. In particular, we examine whether the mismatch explanation is consistent with the incidence of both estrogen receptor positive (ER-positive) and estrogen receptor negative (ER-negative) breast cancers. Because ER-negative breast cancers are typically insensitive to hormones, we predict that these breast cancers may not be associated with higher hormonal exposures characteristic of modern reproductive patterns.

### ER status

One critical difference among breast cancer subtypes that has long been recognized is that of estrogen dependent (ER-positive) versus estrogen independent types (ER-negative). The majority of breast cancers are ER-positive [29]. Breast tumors are clinically defined as ER-positive if a minimum of 10% of cells exhibit ERs (e.g. [30]), meaning that some ER-positive tumors will have 15% ER-positive cells and others may have 95% ER-positive cells. Thus, ER-positive tumors are actually highly diverse with regard to their expression of ER. This diversity may be a result of different stages of progression or may simply be a reflection of pre-existing differences in ER status in normal and pre-cancerous breast cancer tissue [31]. Breast cancers can also be heterogeneous within a tumor, as is clear from results of multiple biopsies in which different degrees of ER positivity are observed in different regions of the tumor [32]. ER-positive breast cancers tend to be easier to treat because they can often be successfully treated with aromatase inhibitors, which block the production of estrogen or the action of estrogen on ERs. Thus, ER status provides a way of categorizing tumors that has proven clinical utility.

There are also two types of ERs, *α* and *β*. The *α* receptor has a greater affinity for estrogen than the *β* receptor, and it appears that invasive tumors have a higher ratio of *α* receptors relative to *β* receptors than is the case in normal breast tissue [33]. Breast epithelial cells also have receptors for progesterone and growth factors such as Her2/Neu. These subtypes will not be covered in this review due to insufficient data availability.

## METHODS

We surveyed the literature and performed several meta-analyses to evaluate whether breast cancer susceptibility by ER status was differentially associated with reproductive factors including parity, age of first reproduction and age of menarche (see Appendix for methods). Age of menopause was not included because of large methodological differences among studies in the calculation of menopausal age. We identified 33 studies that were included in the final analysis, 25 of which were case–control studies and 8 that were cohort studies. Twenty studies were conducted in USA, and 13 were from diverse countries worldwide. The methods for data extraction and analysis are included in the Appendix. The specific cutoffs used to define parity, late age at first birth and late age at menarche varied by study and are listed in each of the figures.

## RESULTS

### Parity

**Figure 1.**
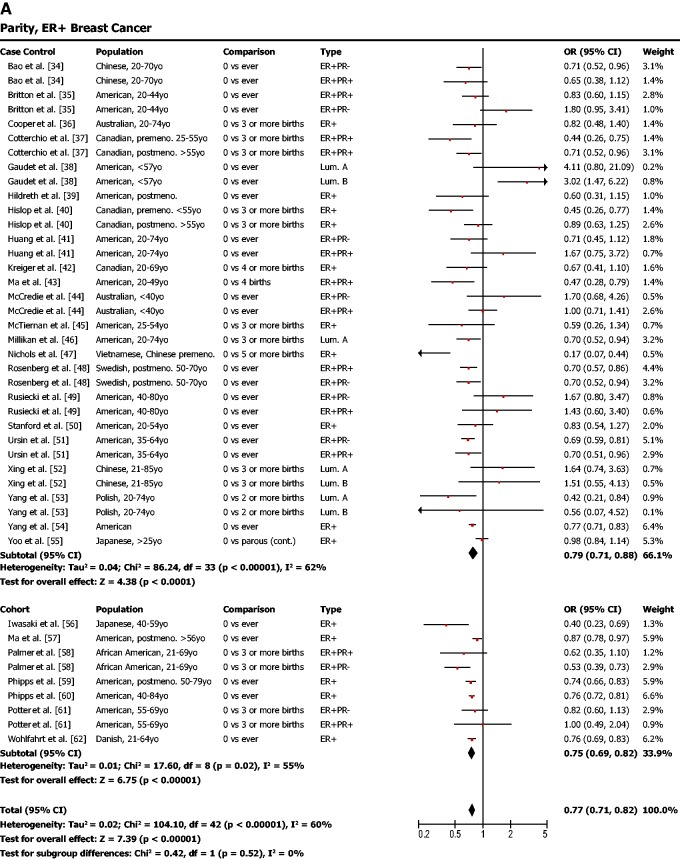

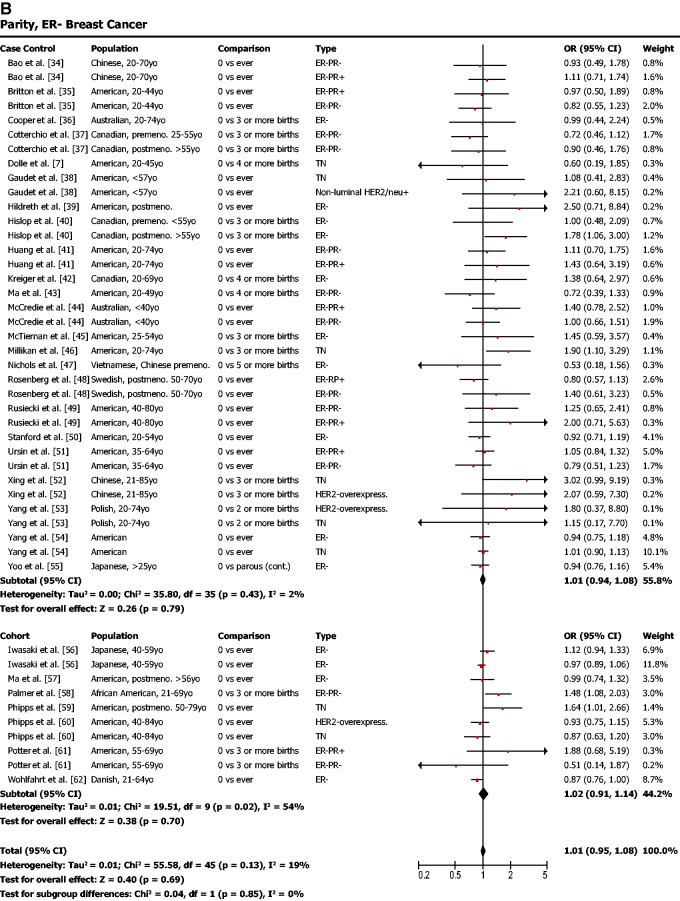
(**a**) Parity is associated with a lower risk of ER-positive breast cancer. (**b**) Parity is not associated with risk of ER-negative breast cancer. OR was calculated using a random effects model to account for heterogeneity of study populations. The red squares and horizontal black lines represent the ORs and 95% CIs for each study. The black diamond and its width represent the overall effect estimate and the 95% CI. The vertical black line represents the null hypothesis (OR of 1). Premeno. = pre-menopausal, Postmeno. = post-menopausal, ER = estrogen receptor, PR = progesterone receptor, TN = triple negative, HER2 = human epidermal growth factor receptor 2, Lum. = luminal

Parity was found to be protective against ER-positive breast cancer (Fig. 1a; odds ratio (OR) = 0.77, 95% confidence interval (CI) = 0.71–0.82, *P* < 0.001) but not protective against ER-negative breast cancer (Fig. 1b; OR = 1.01, 95% CI = 0.95–1.08, *P* = 0.69). In other words, our meta-analysis showed that women who had given birth to one or more children had a lower risk of ER-positive breast cancer but that their risk of ER-negative breast cancer was not affected.

### Age of first birth

**Figure 2.**
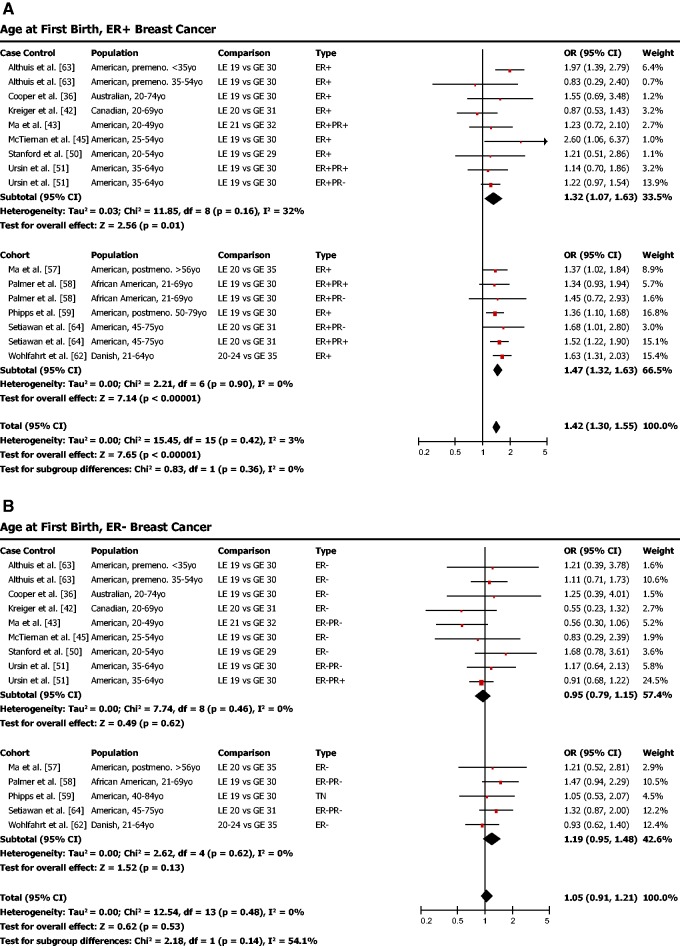
(**a**) Later age of first birth is associated with a higher risk of ER-positive breast cancer. (**b**) Later age of first birth is not associated with risk of ER-negative breast cancer. OR was calculated using a random effects model to account for heterogeneity of study populations. The red squares and horizontal black lines represent the ORs and 95% CIs for each study. The black diamond and its width represent the overall effect estimate and the 95% CI. The vertical black line represents the null hypothesis (OR of 1). Premeno. = pre-menopausal, Postmeno. = post-menopausal, cont. = continuous, ER = estrogen receptor, PR = progesterone receptor, TN = triple negative, HER2 = human epidermal growth factor receptor 2, Lum. = luminal, LE = less than or equal to, GE = greater than or equal to

Our meta-analysis indicated that late age of first birth (after age 30 or 35) was associated with higher odds of ER-positive breast cancer (Fig. 2a; OR = 1.42, 95% CI = 1.30–1.55, *P* < 0.001). ER-negative breast cancer, on the other hand, was not found to be associated with late age of first birth (Fig. 2b; OR = 1.05, 95% CI = 0.91–1.21, *P* = 0.53).

### Age of menarche

**Figure 3.**
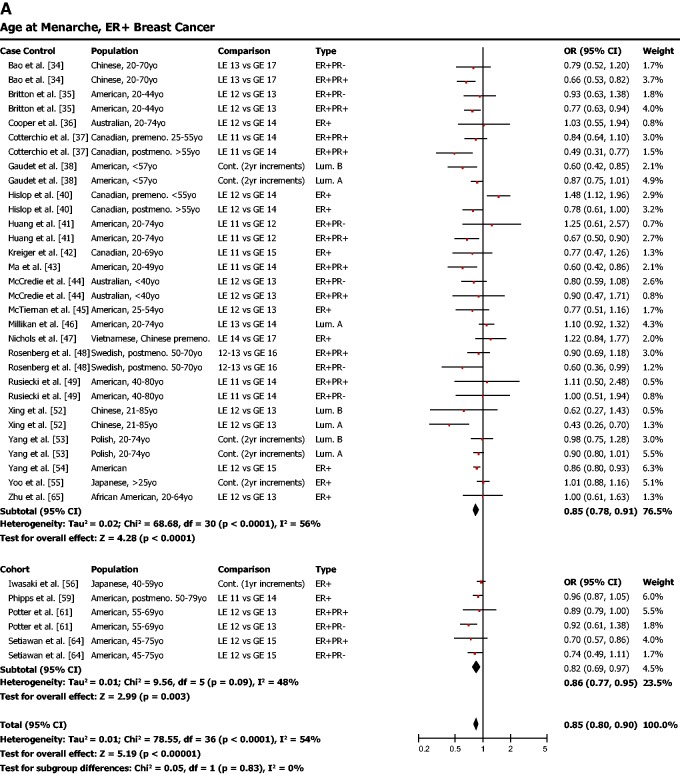

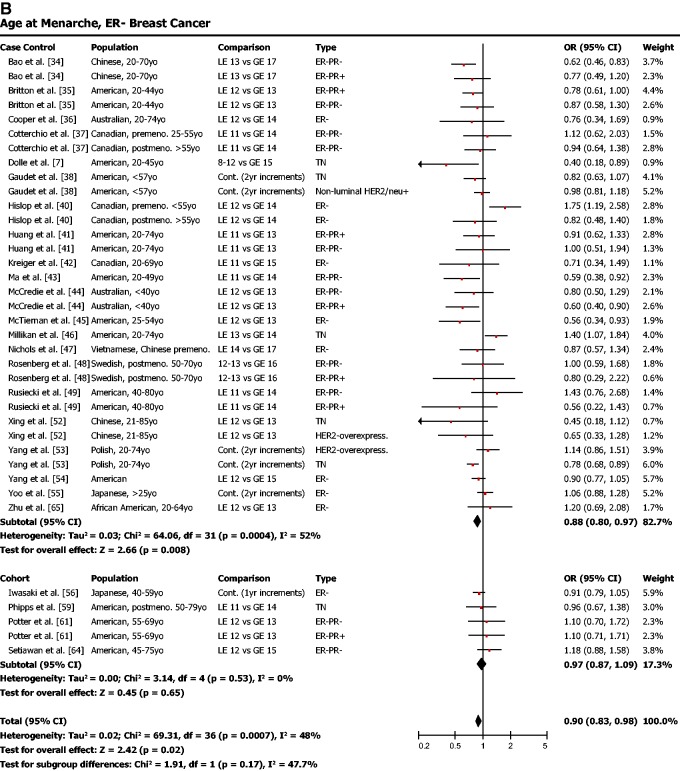
(**a**) Early age of menarche is associated with a higher risk of ER-positive breast cancer. (**b**) Early age of menarche is also significantly associated with risk of ER-negative breast cancer, though the effect is not as strong as for ER-positive breast cancer. OR was calculated using a random effects model to account for heterogeneity of study populations. The red squares and horizontal black lines represent the ORs and 95% CIs for each study. The black diamond and its width represent the overall effect estimate and the 95% CI. The vertical black line represents the null hypothesis (OR of 1). Premeno. = pre-menopausal, Postmeno. = post-menopausal, cont. = continuous, ER = estrogen receptor, PR = progesterone receptor, TN = triple negative, HER2 = human epidermal growth factor receptor 2, Lum. = Luminal, LE = less than or equal to, GE = greater than or equal to

Late age of menarche (typical cutoff around 12 years) was found to be protective against ER-positive breast cancer (Fig. 3a; OR = 0.85, 95% CI = 0.80–0.90, *P* < 0.001). Late menarche was also associated with a lower risk for ER-negative breast cancer (Fig. 3b; OR = 0.90, 95% CI = 0.83–0.98, *P* = 0.02).

In total, each of the three aspects of modern reproductive patterns that we examined in this meta-analysis was significantly associated with ER-positive breast cancer risk at *P* < 0.001. In contrast, risk of ER-negative breast cancer was neither associated with nulliparity nor late age of first birth. For ER-negative breast cancer, only late age of menarche was associated with lower risk, and this effect was weak compared with the protective effect of late menarche on ER-positive breast cancer.

## DISCUSSION

The results of our meta-analysis suggest that modern reproductive patterns are consistent with evolutionary mismatch theory for ER-positive but not ER-negative breast cancer susceptibility. Neither early age at first birth nor higher parity was associated with a decrease in ER-negative breast cancer susceptibility. However, early menarche was associated with increased susceptibility to both ER-positive and ER-negative breast cancer. It is unknown whether early menarche simply increases global risk of breast cancer or whether the role of pubertal timing in breast cancer susceptibility may be more complex, perhaps involving different mechanisms in ER-positive and ER-negative breast cancer susceptibility.

### What mechanisms might underlie susceptibility to ER-negative breast cancer?

Our finding that ER-negative breast cancer susceptibility was not associated with modern reproductive timing and parity puts a new perspective on the generally accepted view that breast cancer susceptibility is associated with an increase in number of cycles and higher levels of cyclic hormone exposure [26, 27]. Our results suggest that ER-negative breast cancer risk may involve mechanisms other than cyclical hormonal exposure. Potential mechanisms underlying ER-negative breast cancers susceptibility include inflammation [66] and insulin resistance [67]. Genetic factors such as *BRCA1* mutations are also associated with hormone-negative breast cancer risk [68], suggesting that physiological processes more likely associated with these *BRCA1* variants, such as lower levels of DNA repair, are potential mechanisms contributing to ER-negative breast cancer susceptibility. Epigenetics may play an important role in ER-negative breast cancer susceptibility as well: Hypermethlyation of *BRCA1* [69] has been found to be associated with triple negative breast cancer but not ER-positive breast cancer. Also, increased methylation of the ER-alpha gene has been found in *BRCA*-linked ER-negative breast cancer [70], identifying a potential mechanism for the reduced expression of the ER-receptor in these tissues. Given that epigenetic changes are also known to regulate ER expression [71], this suggests that epigenetics may be important to understanding susceptibility to both ER-negative and ER-positive breast cancer.

The role of epigenetics in ER expression raises the possibility that the influence of early environment on cancer susceptibility could be mediated by epigenetic changes. Epigenetic changes can occur as a result of physical and social inputs experienced by individuals throughout the life course [72] and have lasting effects [73, 74]. For example, stressful environments can cause epigenetically mediated changes in the functioning of the hypothalamic pituitary adrenal (HPA) axis and glucocorticoid receptors [75], changes in inflammation [76] and even direct effects on tissues such as mammary tissues [77]. Whether epigenetic changes in these processes are specific to ER-negative breast cancer requires further study.

### Applications to other cancers

Like breast cancer, ovarian cancer has both ER-positive and ER-negative subtypes. ER-negative ovarian cancer is associated with lower rates of survival [78] compared with ER-positive ovarian cancer. Breast and ovarian cancer share susceptibility genes such as *BRCA1*/2 [79] and *RAD51C* [80], making it likely that they may share mechanisms underlying cancer risk. Some evidence suggests that the prevalence of ovarian cancer risk factors differ by subtype [81], though this study did not examine whether modern reproductive patterns were associated with ER subtypes in ovarian cancer.

Breast and prostate cancer have a number of similarities with regard to risk factors, tissue physiology and evolutionary history [82]. Like breast cancer, prostate cancer is characterized by both hormone positive and hormone negative subtypes. It has been suggested that certain aspects of modern environments may shape prostate cancer risk. For example, modern dietary conditions may contribute to both breast and prostate cancer risk through similar mechanisms [82]. In addition to modern nutritional conditions, it has been proposed that modern social conditions may contribute to prostate cancer susceptibility through upregulating testosterone production [83]. Whether modern reproductive patterns are risk factors for some subtypes of prostate cancer and not others is an open question.

### Limitations and future directions

In our review and meta-analysis, we found that ER-negative breast cancer susceptibility was not associated with delayed reproduction and low parity, while ER-positive breast cancer was associated with these commonly acknowledged risk factors. ER-negative risk and ER-positive risk were both associated with early menarche. This raises the question of whether early menarche in ER-negative and ER-positive breast cancer susceptibility is due to different underlying mechanisms.

The present approach does not allow us to distinguish between a variety of potential mechanisms that may underlie this differential effect of delayed reproduction and low parity on ER-positive versus ER-negative breast cancer. In future work, we plan to examine potential mechanisms such as upregulated inflammation and epigenetic factors in ER-negative breast cancer susceptibility.

## CONCLUSIONS

Despite general acceptance of the view that cyclical hormone exposure leads to greater breast cancer susceptibility and that factors like parity should therefore be protective, our review of the literature suggests that this view requires revision. Our meta-analysis shows that modern reproductive patterns are strongly associated with ER-positive breast cancer susceptibility but not ER-negative breast cancer susceptibility. These results suggest that modern humans may have higher rates of ER-positive breast cancer (compared with ancestral humans) as a result of current reproductive patterns, including lower parity, later age of first birth and earlier menarche. In contrast, ER-negative breast cancer is associated only with earlier menarche, suggesting that most aspects of modern reproductive patterns are not contributing to ER-negative breast cancer risk. This raises the possibility that ER-negative breast cancer may have different mechanisms underlying cancer initiation and promotion than ER-positive breast cancer. It may be the case that fundamental differences between ER-positive and ER-negative breast cancers with regard to their risk factors have often been overlooked because of the inclusion of ER-negative breast cancers (which are comparatively rare) with ER-positive breast cancers in many studies of breast cancer risk factors.

## APPENDIX: META-ANALYSIS METHODS

### Search strategy

A literature search was conducted in PubMed (up to August 2011) using the following search string: ‘Breast Neoplasms’ AND (‘Receptor, erbB-2’ OR ‘Receptors, Estrogen’ OR ‘Receptors, Progesterone’) AND (‘Parity’ OR ‘Reproductive History’ OR ‘Parturition’ OR ‘Risk Factors’). The citations of relevant articles were also evaluated to identify additional studies that were not identified by the PubMed search. The abstract of each article was used to identify studies that assessed reproductive traits and determined the hormone receptor status of the breast cancer. To be included, the study must have reported a risk ratio (RR), OR or hazard ratio (HR) and 95% CI for various reproductive traits. If multiple studies were published on the same study population, the larger study was selected. Articles that were ultimately reviewed were limited to those with a case–control or cohort study design that had been published in English.

### Data extraction

Relevant data were extracted from each eligible study, including the author’s last name, publication year and country where the study was conducted, measure of association, the 95% CI, the breast cancer subtype and the menopausal status of the subjects if the study population was restricted to a certain subgroup. As breast cancer is a relatively rare disease, the rare disease assumption applies, and RRs, ORs and HRs were assumed to be equivalent. If necessary, the RRs, ORs HRs and 95% CIs were recalculated by taking the inverse of the published value to make the baseline group (reference group) consistent with other studies. If multiple results were reported for the same exposure within the same cohort, the data were prioritized in an effort to prevent the same case population from being included in the meta-analysis more than once. If results from both unadjusted and adjusted analyses were conducted, data from the adjusted analyses were used. If the same study reported associations for multiple breast cancer subtypes (e.g. ER-positive, PR-positive, and ER-positive, PR-negative), both results were included, as both subtypes represent a different group of cases, despite being compared with the same group of controls. For the few studies that conducted both a pooled analysis and one stratified by menopausal status, only the results for the pooled analysis were extracted, in keeping with how the majority of studies handled their analyses. If the study only reported stratified results or was restricted to pre- or post-menopausal women only, the results were included and the status specified in the figure. If multiple ORs were reported for a multi-level categorical comparison (e.g. 0 vs. 1 birth, 0 vs. 2 births and 0 vs. 3 births), only the most extreme comparison was included. For the parity meta-analysis, results were limited to those where the baseline reference group was 0 births (nulliparous women). The age at first birth analyses only included results where there was at least a 10-year difference between early and late age at first birth. A minority of independent studies utilized data from branches within a larger cancer registry (e.g. the Surveillance, Epidemiology and End Results (SEER) Program). Although it is possible that studies that used this registry may include some of the same breast cancer cases in their analyses, we chose to include them as they often used a different control base and modeling approaches. Among the studies included, the age cutoff for early versus late birth was typically 30 or 35, and the age cutoff for early versus late menarche was typically around 12 years.

### Statistical analysis

Given that the studies identified in the literature search originated from many different countries, time periods and populations with varying race/ethnicities and menopausal statuses, it was assumed that there would be great heterogeneity between the various study populations. For this reason, random-effect models were used to calculate summary ORs and 95% CIs.

A random-effect meta-analysis, as described by DerSimonian and Laird [84], was used to combine the results and generate a summary OR and CI. The measures of association were extracted from the publications and log ORs, and standard errors were calculated and imported into the Review Manager Version 5.2 software [85], which was used for the statistical analyses and generation of figures.

While one of the requirements of a meta-analysis is to include one result per study, we felt that it was important to report as many published results as possible in order to display the breadth of the current research. Based on our inclusion criteria, some studies have multiple results included in the meta-analysis dataset, such as results comparing two different breast cancer subtypes to the same control group within the same study. Consequently, this may lead to artificially overconfident summary measures because the model assumes that each result originated from an independent study. The 95% CIs of the summary RRs may be too narrow and the *P*-values are too small.
